# RNA-Seq Analysis of Developing Grains of Wheat to Intrigue Into the Complex Molecular Mechanism of the Heat Stress Response

**DOI:** 10.3389/fpls.2022.904392

**Published:** 2022-06-02

**Authors:** Surinder Paul, Joginder Singh Duhan, Sarika Jaiswal, Ulavappa B. Angadi, Ruchika Sharma, Nishu Raghav, Om Prakash Gupta, Sonia Sheoran, Pradeep Sharma, Rajender Singh, Anil Rai, Gyanendra Pratap Singh, Dinesh Kumar, Mir Asif Iquebal, Ratan Tiwari

**Affiliations:** ^1^Department of Biotechnology, Chaudhary Devi Lal University, Sirsa, India; ^2^Indian Council of Agricultural Research, Indian Institute of Wheat and Barley Research, Karnal, India; ^3^ICAR, National Bureau of Agriculturally Important Microorganisms, Kushmaur, Maunath Bhanjan, India; ^4^Indian Council of Agricultural Research, Indian Agricultural Statistics Research Institute, New Delhi, India; ^5^Department of Biotechnology, Central University of Haryana, Gurgaon, India

**Keywords:** heat stress, grain filling, transcriptomics, gene expression, molecular markers, miRNA targets

## Abstract

Heat stress is one of the significant constraints affecting wheat production worldwide. To ensure food security for ever-increasing world population, improving wheat for heat stress tolerance is needed in the presently drifting climatic conditions. At the molecular level, heat stress tolerance in wheat is governed by a complex interplay of various heat stress-associated genes. We used a comparative transcriptome sequencing approach to study the effect of heat stress (5°C above ambient threshold temperature of 20°C) during grain filling stages in wheat genotype K7903 (Halna). At 7 DPA (days post-anthesis), heat stress treatment was given at four stages: 0, 24, 48, and 120 h. In total, 115,656 wheat genes were identified, including 309 differentially expressed genes (DEGs) involved in many critical processes, such as signal transduction, starch synthetic pathway, antioxidant pathway, and heat stress-responsive conserved and uncharacterized putative genes that play an essential role in maintaining the grain filling rate at the high temperature. A total of 98,412 Simple Sequences Repeats (SSR) were identified from *de novo* transcriptome assembly of wheat and validated. The miRNA target prediction from differential expressed genes was performed by psRNATarget server against 119 mature miRNA. Further, 107,107 variants including 80,936 Single nucleotide polymorphism (SNPs) and 26,171 insertion/deletion (Indels) were also identified in *de novo* transcriptome assembly of wheat and wheat genome Ensembl version 31. The present study enriches our understanding of known heat response mechanisms during the grain filling stage supported by discovery of novel transcripts, microsatellite markers, putative miRNA targets, and genetic variant. This enhances gene functions and regulators, paving the way for improved heat tolerance in wheat varieties, making them more suitable for production in the current climate change scenario.

## Introduction

Wheat (*Triticum aestivum* L.) is the most widely grown cereal crop, accounting for roughly 220 million hectares of agricultural land. Sixty percent of calorie requirement is met by wheat, maize, and rice while wheat alone meets about 25% of protein requirement of the world’s population (Sheoran et al., 2019; Singh et al., 2019). Several abiotic factors affect wheat production, including heat, drought, and salinity. In the present climate scenario, heat stress (HS) is a main constraint affecting the normal growth and productivity of crops, including wheat (Siebert and Ewert, 2014; Fahad et al., 2017), thereby posing a major threat to the global food and nutritional security. Elevated temperature above a threshold level negatively affects all the growth stages. The extent of yield loss depends on the sensitivity of the plant and the phenological/growth stage at which it is exposed to heat stress (Akter and Islam, 2017).

Among all the phenological stages in wheat, the reproductive stage (anthesis and seed development) is considered to be the most sensitive stage for HS (Kumar et al., 2018). It is estimated that during the grain filling stage, per degree temperature rise over and above 18–22°C, decreases grain filling rate and grain yield by 5% and 3–4%, respectively (Gupta et al., 2012). Based on this it was speculated that exposure of 32–38°C temperature during the grain filling stage may cause about 50% yield potential reduction in wheat (Awlachew et al., 2016).

HS tolerance is a very complex process that involves cellular pathway cross-talk. In the early stages of grain filling, HS affects the starch biosynthesis and assimilates translocation leading to poor yield and quality of wheat produce (Farooq et al., 2011; Kumar et al., 2013; Shamloo et al., 2017). Plants modulate their molecular machinery at the transcriptional, post-transcriptional, and epigenetic levels in order to regulate the differential expression of HS-related genes, photosynthetic machinery, cell membrane thermostability, induction of heat shock proteins (HSPs), stress hormones (ABA, ethylene), reactive oxygen species (ROS), compatible solutes (proline, glycine betaine, and sugars) accumulation, and non-coding RNAs synthesis (Farooq et al., 2011; Grover et al., 2013; Goswami et al., 2014; Lal et al., 2021; Zhao et al., 2021).

Many transcriptome studies exist for elucidating molecular mechanisms involving overlapping and distinct regulatory transcriptional mechanism of abiotic stresses response in model plants, as well as in some crops at a specific plant development stage (Coolen et al., 2016; Kang and Yeom, 2018; Cohen and Leach, 2019; Iquebal et al., 2019; Li et al., 2019; Kang et al., 2020; Rangan et al., 2020).

Wheat being a hexaploid with large and complex genome requires modern NGS-based approaches to elucidate tissue and growth stage-specific heat-responsive gene expressions. Wheat transcriptome profiling can reveal the differential gene expression, genome annotations, regulatory factors, molecular markers and expression quantitative trait loci (eQTLs) as well as their sequence variants, controlling the traits of importance (Morozova and Marra, 2008; Berkman et al., 2012; Lal et al., 2021). In wheat, RNA-seq has been adopted mainly to identify new and conserved transcripts associated with abiotic, biotic stress, and nutrient responsive genes (Kumar et al., 2015; Iquebal et al., 2019; Rangan et al., 2020). It is accurate, rapid, and comparatively cheaper and can be applied to non-model plant systems to extract novel genetic information (Unamba et al., 2015). *De novo* transcriptome assembly may be utilized to study the temporal and spatial gene expression of non-model organisms, which is an otherwise difficult task in the absence of complete genome sequence information (Grabherr et al., 2011).

The expression of genes in developing wheat grain is key in determining the ultimate composition of nutritional properties and yield (Henry et al., 2016). Moreover, response to HS is very complex and governed by numerous genes and their regulated expression (Mwadzingeni et al., 2016). Thus, it is of great importance to study the expression of various crucial genes contributing in grain filling and their regulation under HS. Transcriptomics-based expression analysis utilizing RNA-seq is a rapid, sensitive, and accurate way to identify crucial genes, their transcript level variations along with the regulating factors in wheat (Chu et al., 2021). In the present study, we have utilized NGS-based *de novo* assembly approach of RNA-seq to unearth the novel and conserved heat-responsive genes transcripts, transcription factors, gene regulatory network, and their complex interactions in response to heat stress during the initial phases of grain filling stage in heat-tolerant wheat variety K7903 (Halna). Further, the study revealed the discovery of putative molecular markers (SSRs, SNPs, and Indel markers), prediction of transcription factors (TFs), and microRNA and their putative targets. The findings of the study will enrich knowledge-based utilization of heat-tolerant varieties in the crossing block for genetic advancement of wheat.

## Materials and Methods

### Plant Material, Heat Treatment, and Sample Collection

Healthy seeds of a heat stress-tolerant late sown wheat variety, K7903 (Halna), were procured from the germplasm unit of ICAR-Indian Institute of Wheat and Barley Research (IIWBR) Karnal, India. The seeds were surface sterilized with 0.1% HgCl_2,_ followed by three subsequent washing with sterile distilled water. Four wheat seeds were sown in 10-cm pots in the soil in the net house (15 h light, 9 h dark; 40% RH). A total of 18 such pots were sown and labeled. At seven days post-anthesis (DPA), *that is*, approximately 3-month-old wheat plants, 9 out of 18 pots were shifted carefully to Temperature Controlled Phenotyping Facility (TCPF), equipped with automatic sensing of the outside environmental temperature and maintained inside temperature up by +5°C (Sharma et al., 2019). Other environmental factors including humidity, light intensity etc. were maintained similar to outside. The remaining nine pots in the net house served as control. The main tiller spike of each wheat plant in both, control (normal temperature) and TCPF (under HS) conditions was tagged, and the spikelets (wheat grains) were harvested from the middle of the spike at 0-, 24-, 48-, and 120-h intervals after starting from 7 DPA from three main tiller spike and pooled. At each sampling point, four spikelets from the middle of spikes from all the plants were harvested and frozen immediately in liquid nitrogen in a 50 ml sterile plastic tube. The samples taken from controlled or normal conditions were labeled as C1, C2, C5, and C7, while samples from heat stress conditions were labeled as R1, R2, R5, and R7 with two biological replicates.

### RNA Isolation, Library Construction, and Sequencing

The developing wheat grain samples were carefully taken out of the liquid nitrogen without thawing and grounded to a very fine powder using sterile pestle and mortar in liquid nitrogen. Thereafter, total RNA was extracted using RNeasy RNA isolation kit (Qiagen, Germany) according to the manufacturer’s instructions. DNA was removed by digestion with RNase-free DNase (Qiagen, Germany), and RNA was purified and concentrated using RNeasy column (Qiagen, Germany). RNA quality was evaluated by 1% agarose gel electrophoresis for 28 S/18 S rRNA band intensity (2:1) and Agilent 2,100 Bioanalyser and quantified by using NanoDrop Spectrophotometer (Thermo Fisher Scientific, United States). RNA samples were immediately stored in −80°C and subsequently sent for further processing, library construction, and sequencing to AgriGenome Labs Pvt. Ltd., Hyderabad, Telangana, India.

### Pre-processing, *de novo* Assembly, and Expression Analysis

Paired-end (100 bp × 2) read of control and treated samples were generated using Illumina Hiseq 2,500. The quality assessment of reads was performed using FastQC tool.^1^ Cutadapt version 1.8.1 (Martin, 2011) and Sickle version 1.33^2^ were used for removal of adaptors and low-quality reads. Reads having average quality score ≤ 30 were removed. Further, rRNA data from samples were removed with the SILVA database (Quast et al., 2012).^3^ Transcriptome assembly of wheat was performed using *trinity* (at default k-mer length of 25), which uses de Bruijn graphs and dynamic programming algorithm (Haas et al., 2013). Finally, the CD-HIT-EST package was employed to remove redundant sequences from *trinity* assembly (Fu et al., 2012). For the expression analysis, Bowtie 2 (Langmead et al., 2009) was used for alignment of reads, followed by RSEM (RNA-Seq by Expectation–Maximization; Li and Dewey, 2011) for calculation of expression values in the form of FPKM (Fragments per kilobase million reads). RSEM implements improved quantification method having superior accuracy than other methods. Differential gene expression analysis between two biological replicates of each pair was performed using DESeq 1.16.0 program (Anders and Huber, 2010) with parameters, such as Log_2_FC ± 2 and adjusted value of *p* <0.05.

### Homology Search, Functional Characterization, and Gene Ontology

Blastx^4^ program was used for sequence similarity search against NCBI non-redundant protein database with expected threshold e-value 10^−5^ and identity cutoff 40% (Camacho et al., 2009). Contig Annotator Pipeline (CANoPI) was used for gene ontology, pathways analysis of differential expressed genes, and *de novo* transcriptome assembly transcripts whose length was ≥200 bp and FPKM ≥1.0. We also identified transcription factors from the sets of differentially expressed genes using PlantTFDB 4.0 (Jin et al., 2016). The miRNA target prediction from differential expressed genes was performed by psRNATarget server (Dai and Zhao, 2011).

### Identification and Validation of Simple Sequences Repeat Markers

Simple Sequences Repeats were identified from *de novo* transcriptome assembly of wheat using perl script of MISA (MIcroSAtellite identification tool) with default parameters, such as ten repeating units for mononucleotides, six repeating units for dinucleotide, and five for tri, tetra, penta, and hexanucleotides (Thiel et al., 2003). Also, the Primer3 tool was used for generating three sets of primers of identified markers (Untergasser et al., 2012).

From the mined SSRs markers, fifteen were randomly selected for validation in 20 different wheat genotypes panel. Genomic DNA was extracted from seedlings by CTAB method. PCR amplification was carried out in BioRad S1000 Thermocycler in 25 μl reaction mixture containing 2.5 μl 10 × buffer,0.5 μl of 10 mM dNTPs, 0.5 μl of 10 μM each reverse and forward primer, 0.5 μl of Taq polymerase (5 U/μl), 60 ng template DNA and nuclease-free water. PCR amplification was performed using the program: 94°C for 5 min for initial denaturation, 30 cycles of 1 min at 94°C, 50 s at 55–63°C, 72°C for 1 min, and a final amplification of 5 min at 72°C. PCR products were analyzed using 3% agarose gel electrophoresis.

### Identification of Single Nucleotide Polymorphism and Indels

Single Nucleotide Polymorphism (SNP) and Indels (insertion/deletions) were identified from a sample of prior treatment, *that is,* C1 and R1 using two references, *viz.*, *de novo* transcriptome assembly and wheat genome release-31.^5^ Burrows-Wheeler Aligner (BWA; Li and Durbin, 2009) and SAM tools (Li et al., 2009) was used for alignment and calling of variants. Filters, such as read depth coverage >10, quality score > 30, and at least 2 SNPs in 50 bps, were applied to identify significant SNPs and Indels.

### Validation of RNA-seq Analysis by q-PCR

RNA-seq results were confirmed by performing q-PCR of 10 randomly selected differentially expressed transcripts (DETs). cDNA preparation was performed using Superscript® III First-Strand Synthesis System (Invitrogen, UK) following manufacturer’s instructions. Prior to q-PCR, the PCR conditions were optimized by performing successful routine PCR. qPCR mixture contained 1 μl of cDNA (10 ng/μl), 6.0 μl of 2 × SYBR Green Master Mix (Thermo Scientific), and 1 μl (10 μM) each of forward and reverse primer of the selected DET and nuclease-free water to a final 12.0 μl total reaction volume. The quantitative reaction was done on Bio-Rad CFX96™ Real-Time PCR System (Bio-Rad, United States). The qPCR program consisted of 95°C for 5 min, then 40 cycles of 94°C for 15 s, 58°C for 30 s, and 72°C for 30 s and a final melt curve step from 65°C to 95°C with a rise of 0.5°C for 5 s. The relative expression level study of target transcripts was done by comparative 2^−ΔΔCt^ method (Livak and Schmittgen, 2001) using GAPDH as endogenous control.

## Results

### Pre-processing and *de novo* Assembly

**Figure 1:**
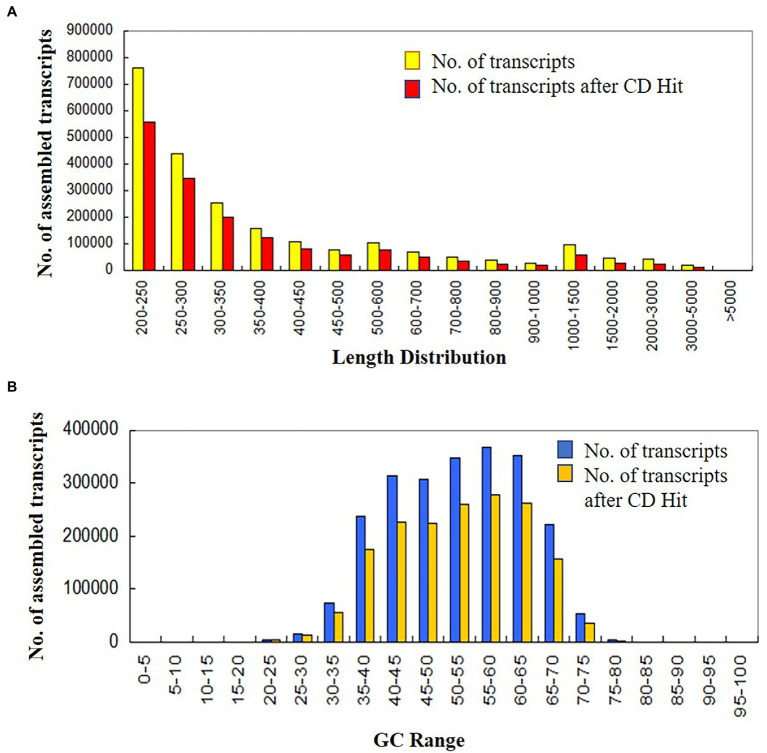
**(A)** Length distribution, **(B)** GC content of assembled transcript in control and treated wheat sample at four different developmental stages (at 0, 24. 48 and 120 h of heat stress treatment).

In this study, a total of 16 samples of control and treated data were generated at four different developmental crop stages *viz.*, 7 days post-anthesis, 0, 24, 48, and 120 h after treatment. Sample names C1, C2, C5, and C7 belonged to the control condition, whereas R1, R2, R5, and R7 represented treated samples. Both control and treated samples had biological replicates. The generated transcriptome data at different developmental crop stages were pre-processed for quality assessment. After trimming and removal of rRNA reads, 253,046,594 (20.5%) poor quality reads were dropped and finally, 979,825,192 (79.47%) high-quality reads were retained (from overall generated data) for *de novo* transcriptome assembly and further downstream analysis. Trinity generated 2,302,239 transcripts with a minimum read length of 200 and GC content of 50.24%. Finally, 1,696,570 transcripts were obtained after removal of redundant sequences with N50 value ≥473 bp, GC content 50.48%, and transcript lengths ranging between 200 to 18,870 bp (Figure 1).

### Functional Characterization and Gene Ontology

**Figure 2:**
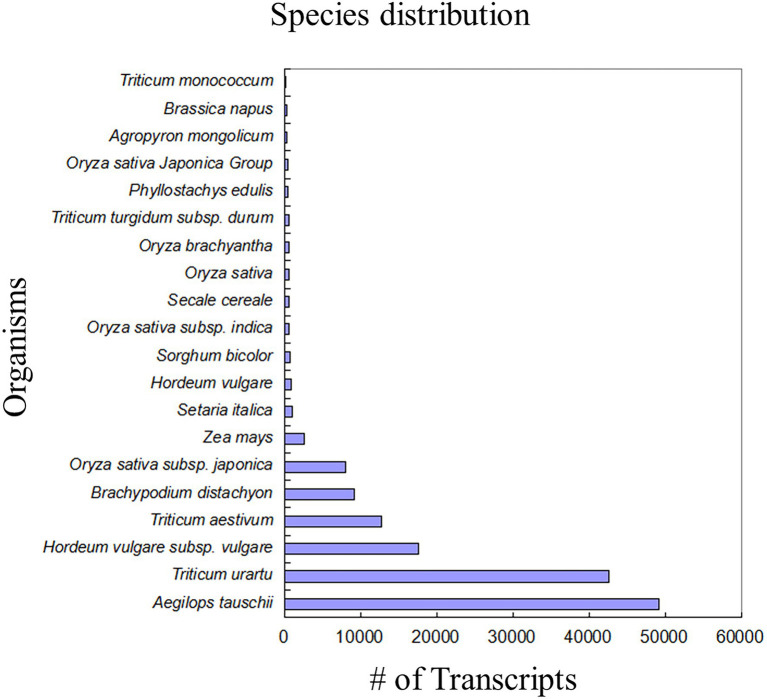
Bar graph representing the species distribution of annotated DEGs. Maximum number of annotated DEGs was represented by *Aegilops tauschii* (49204) followed by *T. uratu* (42504). *Triticum aestivum* falling at the fourth place had approximately 10,000 annotated DEGs.

For homology search and functional annotation, we filtered transcripts having sequence length ≥ 200 bp and FPKM ≥1.0 to finally arrive to a total of 319,879 transcripts for further analyses. The criteria of e-value 10^−5^ and % identity cutoff 40% were employed for searching known sequences against NCBI non-redundant protein database. Out of 319,879 transcripts, 152,842 had similarities with other known proteins in the database. Blastx results showed top hits with *Aegilops tauschii* (49204), *Triticum urartu* (42504), and *Hordeum vulgare* (Figure 2). The identified proteins were further annotated using UniProt database, pathways database, PROSITE, InterProscan, TIGRFAMS, Pfam, PANTHER, and OrthoDB. Finally, a total of 105,127 transcripts were found to have significant Blastx hit and UniProt information (Supplementary File 1).

**Figure 3:**
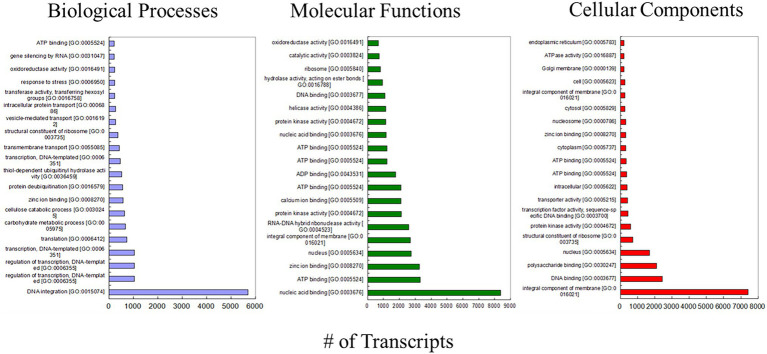
Bar graph depicting gene ontology (GO) classification and enrichment of top 20 GO terms represented under biological (DNA integration: 5688 transcripts), molecular (nucleic acid binding: 8379 transcripts), and cellular components (an integral component of membrane: 7433).

The complete annotations were categorized into Biological Processes (BP), Molecular Functions (MF), and Cellular Components (CC), having 4,345, 3,873, and 2,516 GO terms, respectively. In BP, we found full transcripts represented in DNA integration (5,688 transcripts), while nucleic acid binding (8379) an integral component of membrane (7433) in MF and CC, respectively (Figure 3; Supplementary File 2).

### Abundance Estimation and Differential Expression Analysis

**Figure 4:**
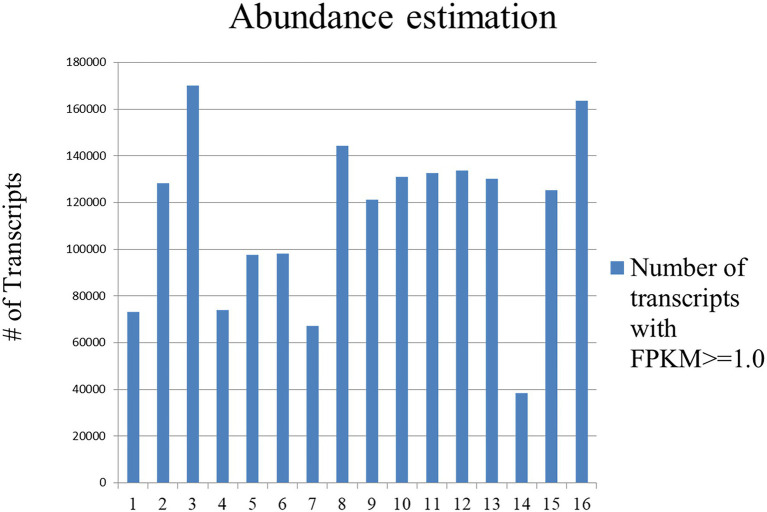
Graphical representations of DEGs with FPKM value ≥1 of 16 libraries prepared in control and treated sample at four different developmental stages. Samples contained 1,696,570 transcripts and ~ 62.48–95.39% (average ~ 83.79%) of reads mapped.

Reads of all the sixteen samples were mapped separately on the *de novo* transcriptome assembly, containing 1,696,570 transcripts and ~ 62.48–95.39% (average ~ 83.79%) of reads mapped. Also, expression values of each sample were calculated in the form of FPKM, to identify differentially expressed genes (Figure 4).

Differential expression analysis was performed and categorized into 3 sets. Set 1 contained the DEGs sets of control samples, such as C1 vs. C2, C2 vs. C5, and C5 vs. C7, whereas set 2 contained treated samples, such as R1 vs. R2, R2 vs. R5, and R5 vs. R7. In the 3^rd^ set, DEGs were of control and treated samples, such as C1 vs. R1, C2 vs. R2, C5 vs. R5, and C7 vs. R7. The DEGs were identified from all the paired sets. In total, 109,455 DEGs were obtained, out of which 95,966 were upregulated and 13,489 were downregulated (Table 1).

**Table 1:** Detailed list of differentially expressed genes obtained in all the three sets.

Sets	Samples name	Upregulated	Downregulated	Total
Set 1	C1 vs. C2	704	12,435	13,139
	C2 vs. C5	46,543	469	47,012
	C5 vs. C7	70	27	97
Set 2	R1 vs. R2	0	0	0
	R2 vs. R5	29	43	72
	R5 vs. R7	4	0	4
Set 3	C1 vs. R1	0	0	0
	C2 vs. R2	48,444	483	48,927
	C5 vs. R5	14	32	46
	C7 vs. R7	158	0	158
	Total	95,966	13,489	109,455

*In total, 109,455 DEGs were obtained, out of which 95,966 were upregulated and 13,489 were downregulated*.

**Figure 5:**
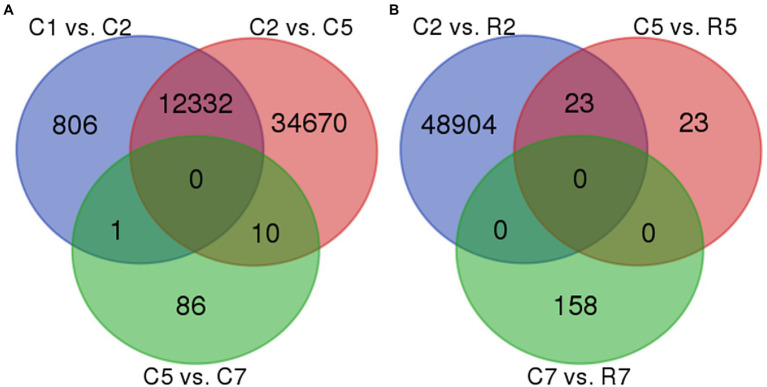
Venn diagram representing two maximum common DEGs in set 1**(A)**-{(C_2_ vs. C_5_) vs. (C_5_ vs. C_7_)} and set 3**(B)**-{(C_2_ vs. R_2_) vs. (C_5_ vs. R_5_)}. 10 DEGs were common in set 1**(A)** and 23 DEGs were common in set 3**(B)**.

In control samples, *that is,* set 1, no common DEGs were found in all the three comparisons, such as C1 vs. C2, C2 vs. C5, and C5 vs. C7. But, several common DEGs were obtained in the comparison set C1 vs. C2 and C2 vs. C5, *that is,* 12,332, while 1 and 10 DEGs were common in comparison “C1 vs. C2 and C5 vs. C7” and “C2 vs. C5 and C5 vs. C7,” respectively. A total of 806, 34,670, and 86 DEGs were found unique in C1 vs. C2, C2 vs. C5, and C5 vs. C7, respectively. Similarly, in Set 2 (treated samples), no common DEGs were obtained. In Set 3, which contains the DEGs of control vs. treated samples, 23 DEGs were found common in between C2 vs. R2 and C5 vs. R5 while 48,904, 23, and 158 DEGs were unique in C2 vs. R2, C5 vs. R5, and C7 vs. R7, respectively (Figure 5).

### Functional Characterization of DEGs

Functional characterization of DEGs was performed using Blastx against NCBI nr database. For identification of transcriptional factors, PlantTFDB v4.0 was used. Transcripts to the tune of 43,861, had hits with other known proteins in the databases (Table 2).

**Table 2:** Number of DEGs in each set that showed similarity with other known proteins in the various databases.

S. No	DEGs	Blast results against NR database	Transcriptional factors (Blast against PlantTFDB v4.0)
1	13,139	5,620 (42.8%)	2064 (15.7%)
2	47,012	17,770 (37.8%)	6,820 (14.5%)
3	97	75 (77.3%)	27 (27.8%)
4	–	–	–
5	72	44 (61.1%)	14 (19.4%)
6	4	3 (75%)	3 (75%)
7	–	–	–
8	48,927	20,272 (41.4)	8,306 (16.9%)
9	46	36 (78.2%)	13 (28.2%)
10	158	41 (25.9%)	47 (29.7%)

*A total of 43,861 transcripts had hits with other known proteins in the databases*.

### MicroRNA Target Prediction

**Figure 6:**
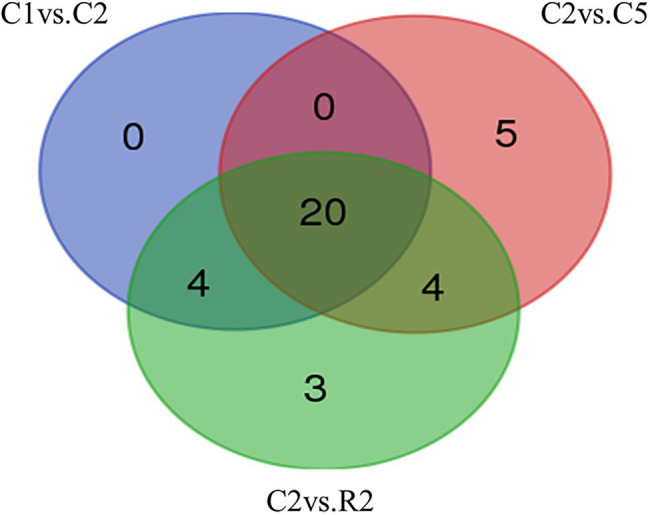
Venn diagram of miRNAs found in DEG sets C_1_ vs. C_2_, C_2_ vs. C_5,_ and C_2_ vs. R_2._ Among all the comparison sets, 20 miRNAs were found to be common.

The miRNA target prediction was made by psRNATarget server for DEGs sets against 119 mature miRNAs of *T. aestivum*. Out of 10 sets, only differential expressed genes of three sets were involved in miRNA target prediction, *that is*, C1 vs. C2, C2 vs. C5, and C2 vs. R2. In set C1 vs. C2, we found 24 miRNAs that targeted 65 transcripts. Maximum 12 transcripts were targeted by “tae-miR1117,” followed by 5 transcripts each in “tae-miR1130b-3p” and “tae-miR1134.” In set C2 vs. C5, 29 miRNAs were found targeting 125 transcripts with a maximum number of transcripts targeted by “tae-miR1117,” *that is,* 51, followed by 9 and 6 transcripts in “tae-miR1128” and “tae-miR1130b-3p,” respectively. Lastly, in DEG set C2 vs. R2, 31 miRNAs were found targeting 154 transcripts and maximum hits were targeted by “tae-miR1117,” *that is,* 55, followed by 9 transcripts each in “tae-miR1130b-3p”and “tae-miR1131” (Supplementary File 5). Twenty miRNAs were found common in C1 vs. C2, C2 vs. C5, and C2 vs. R2, whereas 5 and 3 miRNAs were found unique in C2 vs. C5 and C2 vs. R2, respectively (Figure 6).

### *In silico* Identification of SSR Markers

A total of 98,412 simple sequences repeats (SSRs) were identified from 1,696,570 transcripts of wheat *de novo* transcriptome assembly, while 4,261 repeats were present in compound formation. Out of 98,412 markers, there was abundance of mononucleotides, *that is,* 37,716, followed by di- (27697), tri- (29659), tetra- (2773), penta- (381), and hexa (186)nucleotide repeats (Supplementary File 6). In dinucleotide repeats, we found the maximum number of repeats was GA, *that is,* 4,601, followed by AG (3247) and CT (3101). In the case of trinucleotides, the maximum was GGC repeats, *that is*, 1799, followed by GCG (1487) and GAG (1393; Table 3).

**Table 3:** Details of SSR markers obtained from *de novo* transcriptome assembly as well as unique differential expressed genes of all the stages.

	*De novo* assembly	DEGs (unique DEGs of all sets)
Total number of sequences examined	1,696,570	60,051
Total number of identified SSRs	98,412	2,730
Number of SSR containing sequences	89,321	2,379
Number of sequences containing more than 1 SSR	7,986	292
Number of SSRs present in compound formation	4,261	243
Mono	37,716	668
Di	27,697	832
Tri	29,659	1,154
Tetra	2,773	59
Penta	381	3

*A total of 98,412 simple sequences repeats (SSRs) were identified from 1,696,570 transcripts of wheat de novo transcriptome assembly, while 4,261 repeats were present in compound formation. Out of 98,412 markers, there was abundance of mononucleotides- (37716), followed by di- (27697), tri- (29659), tetra- (2773), penta- (381), and hexa (186) nucleotide repeats*.

### Validation of SSR Markers

**Figure 7:**
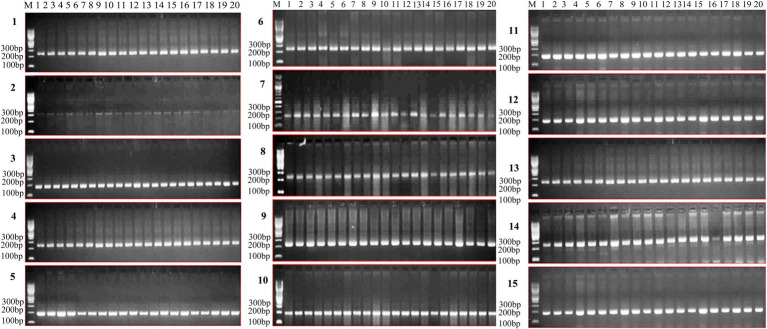
Gel pictures depicting the expression of identified genic SSRs in highly diverse wheat genotypes (1-UP2425, 2-WR544, 3-SONARA64, 4-K7903 (HALNA), 5-WH730, 6-DBW14, 7-RAJ4014, 8-DBW71, 9-AKAW1071, 10-RAJ3765, 11-NIAW34, 12-NW1014, 13-K9465, 14-K9644, 15-HD2733, 16-K9107, 17-PBW502, 18-DBW17, 19-RAJ4083, and 20-WH542) for heat stress.

Twenty highly diverse randomly selected wheat genotypes were used for validation of SSR. PCR primers were designed for randomly selected 20 SSR loci (Table 4), and PCR was amplified using genomic DNA as a template (Figure 7). Such SSR markers are extremely important in crop improvement programs for heat tolerance and wheat breeding not an exception.

**Table 4:** Details of randomly selected SSR markers (thirteen dinucleotide and two trinucleotide repeats) along with their primer sequence and melting temperature ranging from 57.17°C to 60.99°C.

ID	SSR type	Unit	SSR	Primer Pairs (5′-3′)	Tm (°C)
TRINITY_DN252967_c0_g2_i1	Tri	10	(TGT)10	SP-SSR-F1-TGTTGTTGTTGCTTGTGTTGT	57.62
				SP-SSR-R1-TCATTTTATTTGCACATAAACTGCTT	57.17
TRINITY_DN510811_c3_g6_i3	Di	10	(TC)10	SP-SSR-F2-GACACGCACAAACACCCATT	59.61
				SP-SSR-R2-TGACAGAAAAACAGAAAACAGAACA	58.02
TRINITY_DN473094_c8_g3_i1	Di	19	(GA)19	SP-SSR-F3-GTCGTCCTCCTATGCACTCG	59.97
				SP-SSR-R3-CCGCGTGCTGGATTAATTGG	59.97
TRINITY_DN513544_c0_g11_i2	Di	10	(TC)10	SP-SSR-F4-CTGATGATGTTGCGGGCATG	59.97
				SP-SSR-R4-CTCACTGTTGAGCTGCACAA	58.69
TRINITY_DN503444_c13_g12_i1	Di	17	(AG)17	SP-SSR-F5-GGAGAGAGATTGGGCGAGTG	59.89
				SP-SSR-R5-AGGTATTCCCTCCTTCCCCC	60.02
TRINITY_DN438564_c3_g5_i2	Di	12	(TC)12	SP-SSR-F6-GGTATGTACCAGTAGCTATGTGT	57.21
				SP-SSR-R6-CCCATTGTCACCACGGGTAT	59.74
TRINITY_DN456622_c3_g1_i1	Di	10	(GT)10	SP-SSR-F7-TAGTCAGAGACGGGCATCCA	60.03
				SP-SSR-R7-ACACACTTCCACATGTTTATCTGC	59.78
TRINITY_DN523626_c2_g1_i3	Di	17	(TC)17	SP-SSR-F8-TGTCGTGATGCCCAAGTTGT	60.17
				SP-SSR-R8-TGGGTGCCATCCATTGACTC	60.03
TRINITY_DN511017_c1_g2_i7	Di	10	(AG)10	SP-SSR-F9-TGTCAGACTTGCTAGGCGAC	59.75
				SP-SSR-R9-TCAAGACCCACATGACACCT	58.56
TRINITY_DN431119_c0_g4_i2	Di	10	(AG)10	SP-SSR-F10-GTGTTGGGGAACGTAGCAGA	59.96
				SP-SSR-R10-GCGCTTGGATCGGAATCAAC	59.97
TRINITY_DN408106_c2_g1_i1	Di	10	(AG)10	SP-SSR-F11-TCGAAATCGAAAAACATGTCCACA	59.72
				SP-SSR-R11-TGTGCTTTATACTTCGATGTTGTGA	59.07
TRINITY_DN500214_c8_g1_i2	Di	10	(TC)10	SP-SSR-F12-ACGAACTGCTTGGGTGAGTT	59.82
				SP-SSR-R12-TTTGTCCCGGCCTTGTTCTT	60.10
TRINITY_DN459549_c1_g2_i1	Di	11	(TG)11	SP-SSR-F13- GTTGCGTGCGTGTGTGTG	60.64
				SP-SSR-R13-ACGCCTTCTCTTCCCTCTCT	59.96
TRINITY_DN517244_c1_g1_i2	Di	19	(AG)19	SP-SSR-F14-GTTGCATACGAGGAGGGGAC	60.17
				SP-SSR-R14-TCTCCCTCTCCCTCTCCCTC	60.99
TRINITY_DN17441_c0_g1_i1	Tri	13	(CAA)13	SP-SSR-F15-GCCATGTGATGCAGCAACAA	60.03
				SP-SSR-R15-GCTGAGCTTGGATGATACTCT	57.25

### Identification of Single Nucleotide Polymorphism and Indels

Single Nucleotide Polymorphism (SNPs) and insertion/deletion (Indels) were identified from pooled reads of C1 and R1 against *de novo* transcriptome assembly of wheat and wheat genome Ensembl version 31. We found a total of 107,107 variants against the *de novo* transcriptome assembly of wheat, containing 80,936 SNPs and 26,171 Indels. Maximum number of variants (38) were found in transcript ID “TRINITY_DN523265_c0_g1_i3 (WD repeat and FYVE domain-containing protein 3)” and interesting all 38 were SNPs (Supplementary File 7). Similarly, we obtained 110,787 variants against the wheat genome, which contains 99,791 SNPs and 10,996 Indels. Top variants identified from chromosome number 3B, *that is*, 12,977 (11,589 SNPs and 1,388 Indels), followed by 5,788 and 5,771 variants in chromosome 2B and 2A, respectively (Supplementary File 7).

### Validation of RNA-seq Analysis by q-PCR

**Figure 8:**
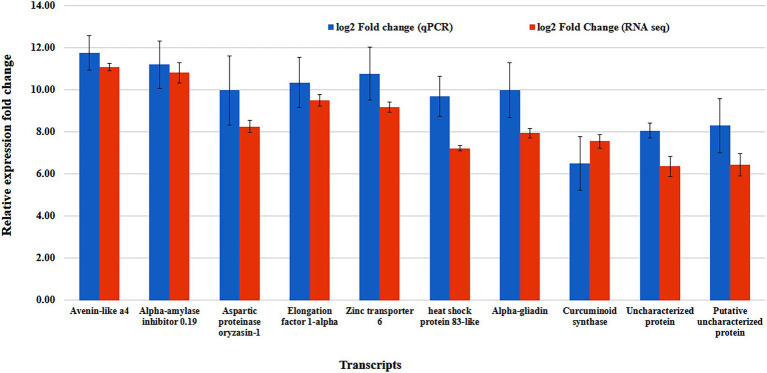
Comparative expression analysis of selected DEGs using RNA-seq and qPCR. Data are means of two independent biological replicates (*p* ≤ 0.05, *n* = 2). Error bars represent the means ± SD (*n* = 2). Accuracy of the RNA-seq data getting confirmation with the qPCR expression pattern results.

The RNA-seq results were verified by performing q-PCR of ten randomly selected differentially expressed transcripts (DETs; Table 5). The qPCR expression pattern results of these DETs were found to follow the same trend as the RNA-seq expression pattern results, confirming the accuracy of the RNA-seq data obtained in this investigation (Figure 8).

**Table 5:** Differentially Expressed Transcripts (DETs) and primers used for qPCR.

Sr. No.	Transcript ID	Primer Pair	Primer Sequence	Tm (°C)	GC %	Amplicon Size (bp)
1	TRINITY_DN521405_c2_g2_i1 (Avenin-like a4)	SP-QF33	TGCCCTTGCTGCTGTCGCATGA	55.44	59.09	137
		SP-QR33	ACAGATGTGGCAGGCAAGCGGT	57.08	58.33	
2	TRINITY_DN483169_c0_g1_i6 (Alpha-amylase inhibitor 0.19)	SP-QF24	AGCCGAGTACGACGCATGGAGCGTT	58.21	60.00	119
		SP-QR24	TGCCATTGCACTGGAGCCTCAGCA	57.21	58.33	
3	TRINITY_DN518599_c1_g1_i2 (Aspartic proteinase oryzasin-1)	SP-QF31	TTAAGCTAGCCCGCTTGTGCCA	52.72	54.55	114
		SP-QR31	ACGGCAAACTAGCGAATCCTGCGT	55.07	54.17	
4	TRINITY_DN326896_c0_g1_i1 (Elongation factor 1-alpha)	SP-QF10	TGAAGGAGCCCTTTCCCATCTCAGCA	55.92	53.85	122
		SP-QR10	ACACGTAGATTCGGGCAAGTCCACCA	56.02	53.85	
5	TRINITY_DN385005_c2_g1_i2 (Zinc transporter 6)	SP-QF12	GTCCCTGCTGTCAGGTGGAGGAATAA	54.07	53.85	125
		SP-QR12	ACGACCGACATGCATCTGAAGTGGA	54.23	52.00	
6	TRINITY_DN425597_c0_g2_i2 (heat shock protein 83-like)	SP-QF15	AAGCCAGAAGACAGGAGCGCAGTT	54.72	54.17	148
		SP-QR15	ACATGGCAGCAAAGAAACACCTGGAG	54.01	50.00	
7	TRINITY_DN490415_c0_g1_i8 (Alpha-gliadin)	SP-QF25	ATTTCCGCGAACTGGGGCAGTTGT	55.47	54.17	119
		SP-QR25	TCCTTCCAACAGCCTCAGCAGCAA	54.81	54.17	
8	TRINITY_DN365152_c1_g1_i1 (Curcuminoid synthase)	SP-QF11	ACGCATTCCGTAGCGCCATTGT	53.39	54.55	143
		SP-QR11	TATATTGTCCAGGATCGGACGCCCT	53.19	52.00	
9	TRINITY_DN472370_c0_g2_i1 (Uncharacterized protein)	SP-QF21	AGAGGCCTTGGGGTTGAAACAACCTT	55.02	50.00	146
		SP-QR21	TTCATCCCGCATCGCCAGTTCTGCTT	56.99	53.85	
10	TRINITY_DN441674_c0_g2_i1 (Putative uncharacterized protein)	SP-QF17	GTTTGTTTTACGGCGTAGCCTCCCGA	55.24	53.85	143
			
		SP-QR17	ACTCAAGCCTCGCTTGGTATTGGGCA	56.65	53.85	

*GC content of the primers was ranged from 50.00 to 60.00% and the size of amplicon was observed to be ranged from 114 to 148*.

## Discussion

Global wheat production is highly vulnerable to climate change and environmental factors, such as heat stress and water deficit, which have severe adverse impact on the wheat crop productivity and quality (Pareek et al., 2020; Rangan et al., 2020). About 57% wheat cultivated area in developing nations is affected by heat stress, thus it can be considered as primary abiotic factor threatening world wheat production and food security (Kosina et al., 2007). Wheat is more sensitive to the heat stress during reproductive (anthesis and post-anthesis) stages and grain filling period which is very crucial for sustaining overall crop yield and quality (Rangan et al., 2020). Heat stress response is very complex mechanism that involves interplay and modulation of numerous biochemical and molecular machinery for sustaining yield and quality (Mwadzingeni et al., 2016). It is of great importance to study the expression of various crucial genes and their regulation in wheat, expressed during grain filling stage under the heat stress. RNA-seq-based next-generation sequencing (NGS) technology provides comparatively economic and efficient way of identifying crucial genes, their transcript level variations along with the regulating factors with high accuracy (Chu et al., 2021).

In the present work, we identified a total of 115,656 genes in heat stress-tolerant wheat genotype, that is, Halna (K7903), exposed to heat stress. Result indicated differential expression of 109,455 DEGs in different sets, out of which 95,966 were upregulated and 13,489 were downregulated (Table 1). The detailed information of DEGs with log2 fold change, adjusted value of p, and their functional characterization is presented in Supplementary File 3. Supplementary File 4 provides the information of transcriptional factors which were obtained in each set. A total of 43,861 transcripts had hits with other known proteins in the databases (Table 2). These DEGs narrowed down to 309 which were involved in many critical processes *viz.*, signal transduction, starch synthetic pathway, antioxidant pathway, and heat stress-responsive conserved and uncharacterized putative genes that play an essential role in maintaining the grain filling rate at high temperature. Categorization of the annotations found their place into Biological Processes (BP), Molecular Functions (MF) and Cellular Components (CC), corresponding to 4,345, 3,873, and 2,516 GO terms, respectively. DNA integration (5,688 transcripts), nucleic acid binding (8379), and integral membrane components (7433) were observed in BP, MF, and CC, respectively (Figure 3; Supplementary File 2). Differential gene expression of transcripts functionally involved in diverse cellular functions is an indicator that the heat stress response in wheat is very complex and it simultaneously regulates different biochemical and molecular pathway sustaining the survival and developmental processes in wheat. Among differentially expressed transcripts, TRINITY_DN521405_c2_g2_i1 (Avenin-like a4) which is cysteine-rich storage proteins expressed during early stages of seed development (3–22 DPA) exclusively in developing endosperm of wheat and its related plant species. Expression of this gene during early grain development plays very significant role in ensuring wheat processing quality (Ma et al., 2013; Chen et al., 2016; Zhang et al., 2018). Jiang et al. (2012) conducted a proteomics-based study and reported that the avenin-like proteins expression is highly upregulated under drought stress and may have an essential role in seed quality. Similarly, Zhang et al. (2020) suggested that avenin-like proteins are multifunctional including role during disease resistance. In our study, we found very high expression of avenin-like proteins which suggests their important role during seed development/grain filling under heat stress. To the best of our knowledge, this is the first report of expression study of avenin-like protein under heat stress in wheat.

TRINITY_DN483169_c0_g1_i6 (Alpha-amylase inhibitor 0.19) are amylase inhibitors, found in cereal grains, including wheat and some legume seeds, which inhibit the target α-amylase enzymes thus protect the starchy seed reserves from degradation into simpler oligosaccharides (Petrucci et al., 1976; Franco et al., 2000; Dombrowski, 2003). Their role in protecting plant amylase inhibitors in biotic stress, particularly insect pests, is well established (Feng et al., 1996; Franco et al., 2000; Rane et al., 2020). Alpha-amylase inhibitors inhibit grain starch degradation and protect the plant against oxidative stress (Hajheidari et al., 2007). Reports suggest that the differential expression of amylase inhibitor at protein level in different cultivars during different grain filling stages play an essential role during grain filling under abiotic stress (Jiang et al., 2012; Zhang et al., 2014). The upregulation of α-amylase inhibitors transcripts under heat stress in our study also confirms their role in heat stress tolerance in wheat during grain filling.

TRINITY_DN518599_c1_g1_i2 (Aspartic proteinase oryzasin-1) are proteolytic enzymes with two highly conserved aspartate residues and play a crucial role in stress responses in plants (Alam et al., 2014). *APs* has been reported to play an important role in protein processing in response to pathogen attack, programmed cell death, water stress, drought, and other environmental stimuli in many plant species at different developmental stages in various plant organs (Runeberg-Roos et al., 1994; De Carvalho et al., 2001; Guevara et al., 2005; Mendieta et al., 2006; Simões et al., 2007; Muñoz et al., 2010; Yao et al., 2012; Rawlings and Salvesen, 2013). Asakura et al. (1995) reported that a rice aspartic proteinase, oryzasin 1 transcripts level was much more abundant from just after flowering stage up to final seed formation suggesting its active role in seed storage protein processing. Yao et al. (2012) reported overexpression of APs under water-deficient conditions in *Zea mays* and their role in drought avoidance through ABA-dependent signaling pathway. They also reported that transgenic *Arabidopsis* overexpressing gene VlAP17, encoding a Group C aspartic protease, enhanced tolerance to salt and drought stress during seed germination, seedling, and maturation. The transgenic Arabidopsis VlAP17 overexpression also increased ABA levels, a reduction in average stomatal aperture size, and elevated expression levels of stress response genes involved in the ABA-dependent pathway well as higher activities of several antioxidases: superoxide dismutase, catalase, and peroxidase. Recently, Sebastián et al. (2020) reported that APA1 encoding AP in known to be highly expressed under water stress in response to salinity, cold, and drought stress. Our study also found that AP expression increases in response to heat stress in plant seed tissue, suggesting their crucial role in response to heat stress.

TRINITY_DN326896_c0_g1_i1 (Elongation factor 1-alpha) Elongation factor 1α- is a multifunctional protein Transcript elongation factors (EFs) play essential role in mediating critical cellular processes related to cellular growth, proliferation, and cell differentiation by interacting with other cellular proteins (Bukovnik et al., 2009; Zheng et al., 2014). Its high expression during heat stress conditions in animals and plants has been reported and thus suggested its essential role in survival under stress conditions (Buckley, 2006; Shamovsky et al., 2006; Bukovnik et al., 2009). They further mentioned its role in heat stress in wheat as accumulation was high in cultivars with better heat tolerance. Zheng et al. (2014) characterized a transcript elongation factor gene in wheat through expression based association analysis, comparing near-isogenic lines and by overexpression in *Arabidopsis* and reported its role in regulating yield-related traits involved in vegetative growth and reproductive development. In our study, the upregulated expression of the EF also suggests its role during the grain filling stage in wheat. Metabolic proteins of wheat, mainly involved in glycolysis, carbohydrate metabolism, and the stress response, show differential expression profiles in response to elevated temperature conditions (Laino et al., 2010).

TRINITY_DN490415_c0_g1_i8 (α-gliadin) was found to be upregulated in the present study. Yang et al. (2011) has reported that the expression of many storage proteins is under heat or water deficit, *viz.*, α-gliadin, γ-gliadin, low molecular weight glutenin, and globulins in the seeds altered. Further, the abundance of glidins was found to increase under heat stress from anthesis up to 10 DPA. Du Pont et al. (2006) also studied that amount of α-gliadins increases in response to elevated temperature conditions during endosperm development in wheat. Our results are in agreement of the above studies.

TRINITY_DN425597_c0_g2_i2 (heat shock protein 83-like) belongs to the heat shock protein group, which are well known for their protective role in response to elevated temperatures in almost all organisms, including plants. These proteins perform multiple functions, such as protein folding, translocation across membranes, facilitation of protein–protein interaction, and preventing protein aggregation and regulation of synthesis of other stress-related genes during heat stress conditions (Åkerfelt et al., 2010; Roy et al., 2019). Conner et al. (1990) reported the heat-inducible expression of AtHS83 in *Arabidopsis thaliana*. Felsheim and Das (1992) reported the differential expression of HSP83 member genes in response to heat stress and photoperiod duration in cotyledons. Disruption of genes encoding HSP83 has been shown to have detrimental effects on the growth and survival in yeast at high temperature (Borkovich et al., 1989). Our results also agree with the above study and we can conclude that heat shock proteins also have an essential role in wheat during heat stress.

TRINITY_DN385005_c2_g1_i2 (Zinc transporter), a transmembrane zinc transporter is reported to confer heat tolerance during grain filling stage by maintaining optimum cellular zinc concentration required for the proper working of an enzyme related to stress tolerance carbohydrate metabolism and normal seed filling process under heat stress (Kobayashi et al., 1998). They also reported zinc transporter’s role in normal flowering and fruit development. Moreover, the Zn is crucial for plant growth and development as it is required by various enzymes involved in carbohydrate metabolism, cell membrane stability and integrity, protein synthesis, auxin synthesis regulation, ribosomal stabilization, and cytochrome synthesis (Marschner, 1995). Zn also plays role in the stress tolerance mechanism of plants by regulating gene expression of various stress-related genes (Cakmak, 2000). Homeostasis in cellular zinc concentration improves the plant tolerance to heat and salt stress in various plant species by improving the water uptake and transport (Peck and McDonald, 2010; Tavallali et al., 2010; Disante et al., 2011). Sarwar et al. (2019) reported the role of exogenously applied zinc and other micronutrients in heat stress alleviation in cotton. Specific Zn transporters maintain the optimum cellular concentration through influx, efflux, and compartmentalization. Thus, we can also suggest that upregulation in gene expression of zinc transporter 6 in our study contribute to heat tolerance in wheat.

Therefore, the transcripts validated randomly using q-PCR in the present study are multifunctional. Most of them are reported to be overexpressed in response to multiple stress conditions including heat, drought, pathogen, or insect attack. Some genes are very crucial for maintaining seed quality parameter *viz.,* number of different seed-specific protein content and carbohydrate metabolism specifically under heat stress. Some transcripts also play protective role during heat stress ensuring proper protein folding, protein translocation, and enzyme functioning. Other transcripts play significant role in regulating seed developmental processes under heat stress by regulating molecular processes including gene expression, protein synthesis etc. Further, these transcripts help in better understanding of complex molecular and biochemical mechanism of heat stress response in wheat.

In addition, we also identified SSRs from wheat *de novo* transcriptome assembly and characterized them. The repeat pattern of identified SSRs indicated an abundance of mononucleotides followed by di, tri, tetra, penta, and hexanucleotide repeats (Supplementary File 6). In dinucleotide repeats, we found the maximum number of repeats as GA followed by AG and CT. In the case of trinucleotides, GGC repeats were most abundant followed by GCG and GAG (Table 3). Additionally, we found a total of about one hundred thousand variants against the *de novo* transcriptome assembly of wheat, containing SNPs and Indels. Thirty-eight variants were reported in transcript ID “TRINITY_DN523265_c0_g1_i3 representing WD repeat and FYVE domain-containing protein 3” incidentally all of them being SNPs (Supplementary File 7). Similarly, we obtained one hundred and ten thousand variants against the wheat genome, containing SNPs and Indels. Maximum variants were identified from chromosome number 3B followed by chromosomes 2B and 2A (Supplementary File 7). Being high throughput and high density in nature coupled with widespread distribution throughout the genome, SNP markers are popular and important for their utilization in population genetic diversity studies, gene mapping, and high-density linkage map construction (Unterseer et al., 2014; Carrillo-Perdomo et al., 2020). Therefore, the genic-SSR markers and SNP markers reported in this study along with previously developed markers from other laboratories will be a powerful resource for molecular taxonomic studies and construction of a reference molecular map of the wheat genome. Since such genic-SSR and SNP markers represent the gene-rich regions of the genome, many of these can be utilized in marker-assisted breeding (Dutta et al., 2011). Also because of conservation of genic sequences, these markers have a greater chance of transferability across species, compared to genomic SSR markers which show high polymorphism but are less conserved between species (Wang et al., 2021).

The miRNAs are known as key players in regulating plant gene expression *via* targeting important genes involved in growth and developmental during heat stress (Xin et al., 2010; Kumar et al., 2015). DEGs sets using 119 mature miRNAs were subjected to miRNA target prediction for *T. aestivum*. Out of total ten sets, only differential expressed genes of three sets were involved in miRNA target prediction. miRNAs were found with a maximum number of transcripts targeted by “tae-miR1117.” Also in DEG set C2 vs. R2, 31 miRNAs were found with maximum hits were targeted by “tae-miR1117” (Supplementary File 5). Common miRNAs in C1 vs.C2, C2 vs. C5, and C2 vs. R2 were 20 in number while there were only 5 and 3 miRNAs that were found unique in C2 vs. C5 and C2 vs. R2, respectively (Figure 6). These tiny miRNAs are very crucial to modulate the physiological, biochemical, and molecular responses by targeting key genes during heat stress.

Taking together, the present study enriches our current understanding of known heat response mechanisms during the grain filling stage in wheat supported by discovery of novel transcripts, microsatellite markers, putative miRNA targets, and genetic variant. Further work on functional validation of these genes, miRNAs, SSRs, and SNPs could pave the way for developing improved heat tolerance in wheat varieties, making them more suitable for production in the current climate change scenario.

## Conclusion

Climatic change has affected the quantity and quality of major food crops and thus poses a significant threat to global food security. Initial few days of grain filling are crucial for the net yield of the crop. Heat stress during this period drastically reduces the overall yield. Our *de novo* transcriptomics-based study of the heat stress-tolerant wheat genotype K7903 (Halna) reveals that the upregulation of key genes during initial grain filling stages might be playing crucial role in heat tolerance mechanism. K7903 (Halna) might have genetic and epigenetic (miRNA expression) control mechanism which might be contributing toward avoidance of the detrimental effect of heat stress and thereby maintaining yield and grain quality. The significant findings could further be functionally validated using reverse genetic tools either in wheat or any other model plants which might assist molecular breeders to design suitable strategies to develop heat-tolerant wheat genotypes. In this study, we identified heat stress-responsive miRNA and their target genes, which have critical role in gene regulation during grain filling under heat stress conditions. This valuable information will enrich our knowledge about involvement of different key genes and their expression pattern regulating the grain filling process when exposed to high temperature conditions. This knowledge can be further utilized in identification, characterization, and breeding strategies to develop heat stress-tolerant wheat varieties.

## Data Availability Statement

The datasets presented in this study can be found in online repositories. The names of the repository/repositories and accession number(s) can be found at: https://www.ncbi.nlm.nih.gov/, PRJNA813970, SAMN26531246, SAMN26531247, SAMN26531249, SAMN26531250, SAMN26531251, SAMN26531252, SAMN26531253, SRR18273340, SRR18273339, SRR18273338, SRR18273337, SRR18273336, SRR18273335, SRR18273334, SRR18273333.

## Author Contributions

RT and JD conceived the theme of study. SP, SJ, UA, MI, and RS analyzed the data. SP, JD, MI, PS, RS, and RT drafted the manuscript. SP, NR, and SS conducted the field work. OG and DK were instrumental in formatting the tables and graphs. RT, JD, AR, GS, DK, and MI provided overall guidance and edited the manuscript. All authors read and approved the final version of the manuscript.

## Funding

This work was supported by CABin grant (F. No. Agril. Edn.4–1/2013-A&P) of ICAR-IASRI, New Delhi in alignment with in-house project of ICAR-IIWBR, Karnal.

## Conflict of Interest

The authors declare that the research was conducted in the absence of any commercial or financial relationships that could be construed as a potential conflict of interest.

## Publisher’s Note

All claims expressed in this article are solely those of the authors and do not necessarily represent those of their affiliated organizations, or those of the publisher, the editors and the reviewers. Any product that may be evaluated in this article, or claim that may be made by its manufacturer, is not guaranteed or endorsed by the publisher.

## Footnotes

^1^ http://www.bioinformatics.babraham.ac.uk/projects/fastqc/

^2^ https://github.com/najoshi/sickle

^3^ https://www.arb-silva.de/

^4^ https://blast.ncbi.nlm.nih.gov/Blast.cgi

^5^ ftp://ftp.ensemblgenomes.org/pub/plants/release-31/fasta/triticum_aestivum/dna/
